# Horizontal Gene Transfer of an IncP1 Plasmid to Soil Bacterial Community Introduced by *Escherichia coli* through Manure Amendment in Soil Microcosms

**DOI:** 10.1021/acs.est.2c02686

**Published:** 2022-07-27

**Authors:** Gonçalo Macedo, Asmus K. Olesen, Lorrie Maccario, Lucia Hernandez Leal, Peter v. d. Maas, Dick Heederik, Dik Mevius, Søren J. Sørensen, Heike Schmitt

**Affiliations:** † Department of Infectious Diseases and Immunology, Faculty of Veterinary Medicine, Utrecht University, Yalelaan 1, 3584 CL Utrecht, The Netherlands; ‡ Wetsus, European Centre of Excellence for Sustainable Water Technology, Oostergoweg 9, 8911 MA Leeuwarden, The Netherlands; § Department of Biology, University of Copenhagen, Copenhagen 2100, Denmark; ∥ Van Hall Larenstein, University of Applied Sciences, Agora 1, 8901 BV Leeuwarden, The Netherlands; ⊥ Institute for Risk Assessment Sciences, Utrecht University, Yalelaan 2, 3584 CM Utrecht, The Netherlands; # Department of Bacteriology and Epidemiology, Wageningen Bioveterinary Research, Houtribweg 39, 8221 RA Lelystad, The Netherlands; ∇ Centre for Infectious Disease Control, National Institute for Public Health and the Environment (RIVM), Antonie van Leeuwenhoeklaan 9, 3721 MA Bilthoven, The Netherlands

**Keywords:** lateral gene transfer, antibiotic resistance gene, soil microbiome, mating, cattle manure

## Abstract

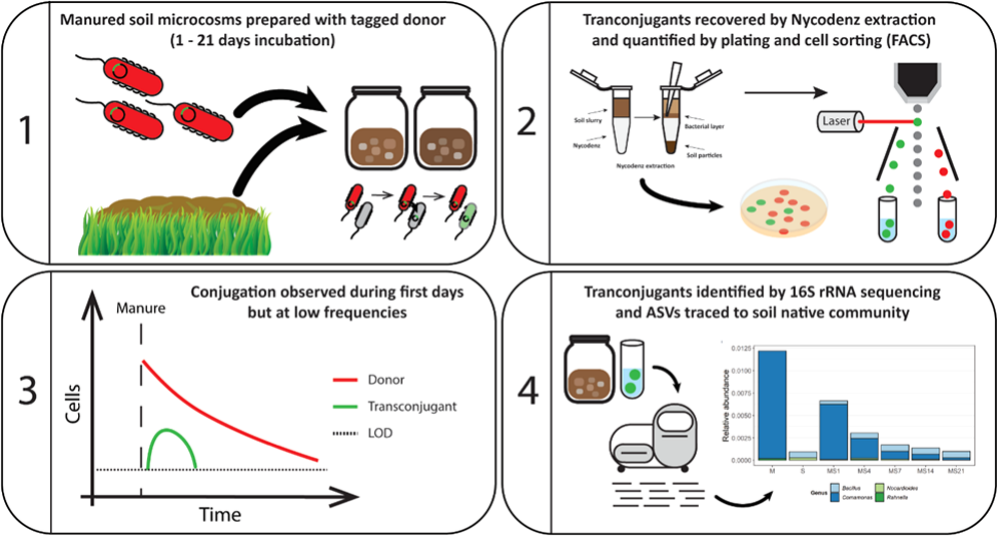

The quantification and identification of new plasmid-acquiring bacteria in representative mating conditions is critical to characterize the risk of horizontal gene transfer in the environment. This study aimed to quantify conjugation events resulting from manure application to soils and identify the transconjugants resulting from these events. Conjugation was quantified at multiple time points by plating and flow cytometry, and the transconjugants were recovered by fluorescence-activated cell sorting and identified by 16S rRNA sequencing. Overall, transconjugants were only observed within the first 4 days after manure application and at values close to the detection limits of this experimental system (1.00–2.49 log CFU/g of manured soil, ranging between 10^–5^ and 10^–4^ transconjugants-to-donor ratios). In the pool of recovered transconjugants, we found amplicon sequence variants (ASVs) of genera whose origin was traced to soils (*Bacillus* and *Nocardioides*) and manure (*Comamonas* and *Rahnella*). This work showed that gene transfer from fecal to soil bacteria occurred despite the less-than-optimal conditions faced by manure bacteria when transferred to soils, but these events were rare, mainly happened shortly after manure application, and the plasmid did not colonize the soil community. This study provides important information to determine the risks of AMR spread via manure application.

## Abstract 2

Our work shows how a common farm practice (i.e., manure application) led to plasmid uptake by native soil bacteria and contextualizes conjugation rates with nationwide data.

## Introduction

Antimicrobial resistance (AMR) has been pinpointed as one of the most significant global public health challenges.^1^ Horizontal gene transfer (HGT) of resistance genes is of particular concern because it drives bacterial evolution^2^ and is connected with the rise of AMR.^3−5^ Plasmid-mediated gene transfer by conjugation is considered a major HGT mechanism.^6−8^

Agricultural application of manure as organic fertilizer results in the introduction of fecal bacteria, their plasmids, and antimicrobial resistance genes (ARGs) into soils.^9,10^ Plasmid conjugation in soils has been extensively studied,^11^ and plasmid transfer from fecal bacteria to soil bacteria has been observed,^12−14^ but the quantification of these transfer events in soils is challenging, and the identity of the new plasmid hosts is often unknown. The quantification of AMR-relevant plasmid transfer events together with the identification of the new plasmid hosts in representative mating conditions is critical to characterize the risk of HGT in the environment.

While mating under close contact (e.g., on filters) is a method often used for quantification of HGT, microcosm systems represent a better approximation of the natural environment as they preserve soil structure. Microcosm setups have indeed shown HGT potential, including studies using *Escherichia coli* as donor, in which the transconjugant abundance reached 10^2^ and 10^3^ CFU/g soil (10^–2^ and 10^–4^ transconjugants-to-donor ratios; T/D).^15,16^ Top et al. (1990) reported 10^2^ transconjugant CFU/g soil (10^–4^ T/D) with an IncP1 plasmid in nonsterile soil, but only when nutrients were added. Notably, manure application to soil provides nutrients and a high density of potential ARG-carrying bacteria, thus creating favorable HGT conditions.^14^ On the other hand, when introduced to soils, fecal bacteria concentrations tend to decline,^18,19^ limiting the time span for potential ARG transfer to soil bacteria. However, culture-dependent microcosm studies such as the ones mentioned above often are operated under unrealistic conditions (e.g., low community diversity^15,16,20^ and high bacterial densities of donors or recipients^21−24^).

Furthermore, culture-based studies are limited to the growth of the hosts, recipients, or both and therefore are not able to unravel transconjugants that are unculturable. To circumvent this challenge, culture-independent methods have been developed to estimate HGT potential (e.g., metagenomics, correlation analysis), but the link between ARGs or plasmids to their hosts is still limited. Alternatively, the use of reporter gene platforms with known donors has provided good results when assessing the recipient range in agricultural soils,^4,25−30^ and using this approach, it was shown that exposure to manure increased the plasmid uptake potential from the soil bacterial community.^27^ However, all of these studies resorted to filter matings.

Recently, the fate of an ARG-carrying plasmid was assessed in greenhouse soil microcosms^31^ under more realistic conditions, showing that reporter gene studies can indeed be applied, but manure was not added to the soils. Therefore, the role of manure as a source of, for example, resistance genes that could be transferred to environmental bacteria through HGT in soils remains unstudied, especially in conditions that resemble environmental conditions in terms of temperature, incubation period, mating matrix, and recipient community diversity.

The main goal of this study was to quantify conjugation events resulting from manuring of soils under conditions more representative of environmental conditions than filter matings. Additionally, we identified the hosts resulting from these conjugation events and, so, provided important information to determine the risks of AMR spread via manure application on land.

## Materials and Methods

### Donor Strain and Plasmid Characteristics

In this study, manured soil microcosms were spiked with*Escherichia coli* MG1655 (chromosomally tagged by *lacIq*-P*lpp*-*mCherry*), carrying pKJK5 (IncP1; tagged with P*lac*-*gfpmut3B*), which was used as donor. This combination of host and vector was also used in previous works studying the recipient bacterial community of soils,^25,27−31^ and details on the genetic surroundings of the inserted gene cassettes can be found in the Supporting Information (Supporting Figure 1). Briefly, the donor cells contain a conjugative plasmid tagged with the green fluorescent protein gene (*gfpmut3B*) downstream from a *LacI* repressible promoter. The donor chromosome encodes *LacI*, which represses the expression of *gfpmut3B* while the plasmid is in the donor. During conjugation, the plasmid is transferred from the donor cells to the recipients, which become transconjugants. Because transconjugants do not encode the *LacI*, the expression of *gfpmut3B* is not repressed in the transconjugant cells, and these cells consequently fluoresce green.^6^*E. coli* was taken as a representative Gram-negative taxon of relevance for the introduction of manure-borne pathogens into soil.

The donor strains were grown aerobically with agitation, at 37 °C, for 3.5h from a fresh dilution of an overnight growth culture (defined by a growth curve) in LB medium supplemented with kanamycin (100 μg/mL). The bacterial cells were harvested by centrifugation at 10,000*g* for 10 min, and the pellets were washed twice and resuspended in 0.9% sterile saline solution. The resulting concentration of donor inoculants was confirmed by plating.

### Manured Soil Microcosms

Cattle manure and grassland soil samples (sandy loam texture) were collected from an experimental farm of the University of Copenhagen in Taastrup (Denmark) in September 2019. The soil properties have been reported in previous publications,^32^ and it is classified as a sandy loam (16% clay, 15% silt, and 69% sand), with pH 7.2 and 1.5 g total N/kg soil. The manure had pH 9.1 and contained 1.7 and 1.6 g/kg (dry weight) of P and K, respectively. The soil and manure samples were stored (up to two months) until used, at 4 °C, to minimize potential changes in the bacterial community structure and activity.

Manured soil microcosm series were prepared in 50 mL tubes containing 15 g of soil (total weight), in four replicates, and were incubated either at 15 or 30 °C, in the dark, for up to 21 days. The donor strain was spiked to manure so that initial theoretical donor concentrations would correspond to 10^7^ CFU/g soil, and the spiked manure was then immediately applied to soils. The amount of manure used approximately corresponds to a general manure application for arable soils. Each microcosm series were prepared by combining the total amount of soil to be used in the replicates of that series (15 g × 4 replicates) with the spiked manure (40 mg/g, fresh weight), and after proper mixing, the manured soil was distributed into the 50 mL tubes containing 15 g of soil that were later incubated. The homogeneity of the donor spiking on the four microcosm replicates was confirmed by the bacterial donor counts, which were prepared from 5 g of the microcosms and yielded concentrations of 6.54–6.92 log CFUs/g, and by estimating the abundance of the genus “*Escherichia-Shigella*” in MS1 (ranging between 25–29%; values in table “rrs_MS_otu” and its graphical correspondence in Supporting Figure 2, both in the Supporting Information). Both outcomes evidenced that the deviation between soil replicates was minor. The water holding capacity of the microcosms was adjusted to 60%, and the tubes were not tightly closed to allow gas exchange throughout incubation. To compensate for the weight loss due to evaporation, the microcosms were regularly irrigated with sterile distilled water.

Destructive sampling occurred before manure amendment to soils (soil, S) and at specific time points after manure application, corresponding to days 1, 4, 7, 14, and 21 (manured soil, MS1 to MS21). Part of each replicate was stored at −20 °C for DNA extraction of the total bacterial community, and another part was used for Nycodenz extraction of the bacterial communities. Control microcosms (i.e., without donor inoculation), were prepared and incubated under the same conditions. Additionally, aliquots of the original manure and soil aliquots were also stored at −20 °C for DNA extraction.

### Nycodenz Extraction and Recovery of Transconjugants

Nycodenz density gradient separation was used to extract the bacterial communities and proceeded as described by Klümper et al.,^33^ with reagent volumes adapted to match the used 5 g of the microcosms. The Nycodenz extracts were stored at near-zero temperatures (on ice and at 4 °C) until used for enumeration or cell sorting.

To enumerate donors and transconjugants in the Nycodenz extract, serial dilutions were prepared, and 100 μL of each dilution was plated on LB agar, containing kanamycin (100 μg/mL), trimethoprim (32 μg/mL), and sulfamethoxazole (128 μg/mL) to guarantee that cells without the tagged pKJK5 would not grow. Nystatin (20 μg/mL) was also added to prevent fungal growth in the plates. These antibiotics were chosen because the tagged pKJK5 carried the respective ARGs (Supporting Figure 1). Additionally, the phenotypic resistance conferred by the plasmid to the donor had been confirmed by an antimicrobial susceptibility test (interpreted according to EUCAST guidelines). The plates were incubated at 30 °C, for 24 h, and colonies were observed and counted using a Dark Reader Transilluminator (Clare Chemical Research) for GFP excitation; total cells and green fluorescing cells correspond to donor and transconjugants. Because low temperatures favor the maturation of GFP,^34^ the plates were re-counted after a 24 h incubation at 4 °C, thus confirming the results obtained directly after incubation. The plating of the microcosm control series confirmed that no background was observable in plates with the mentioned antimicrobials. A schematic of the sample processing workflow is available in the Supporting Information (Supporting Figure 3).

### Flow Cytometry and Transconjugant Sorting

Cells obtained with Nycodenz extraction were analyzed and sorted using a FACSAria IIIU (BD Biosciences) equipped with the BD FACSDiva software v8.0.3 (BD Biosciences). A 70 μm nozzle was used with a sheath pressure of 70 PSI. To detect bacterial cells, both forward scatter (FSC) and side scatter (SSC) were used, and their threshold was lowered to the minimum of 200 in signal height. The green fluorescence of GFP was excited by a 488 nm laser (20 mW) and detected using a 530/30 nm bandpass filter. The red fluorescence of mCherry was excited using a 561 nm laser (50 mW) and detected using a 610/20 nm bandpass filter. The gating was made so that a double logarithmic bivariate plot with FSC-Area and SSC-Area was used to detect events in the bacteria’s size and complexity. These events were forwarded to a double logarithmic bivariate plot with green fluorescence intensity and red fluorescence intensity, in which transconjugant events were detected as only green fluorescent and donor cells as red fluorescent. Before being loaded, the samples were diluted in PBS until an event rate of ∼3000 events/s was obtained. For sorting, the purity precision settings were used.

Due to the low number of overall transconjugants observed (see the Results section and Figure 2), the expected required sorting time would be excessively high (approx. 20h/replicate). Adding to the amount of time needed for sorting, the longer the period spent in sorting, the higher the chance of errors. Therefore, for practical reasons, either 30 or 300 transconjugants (for the 15 or 30 °C microcosm series) were collected from time point MS1 for each microcosm replicate, resulting in a total of 1320 transconjugants collected. To maintain a sufficient number of transconjugant cells for subsequent sequencing, the sorted cells were incubated in sterile 10% soil extract for 3 days, at a corresponding microcosm series temperature, and to avoid excessive growth bias. The soil extract was obtained from the same soil used for the microcosm experiments, using a previously described method.^26^ After the 3-day incubation, because no observable signs of growth were visible to the eye, sterile 10% TSB (tryptone-soy broth) was added to the sorted cells, and they were incubated for one additional day at the same temperature as before. After this period, only the DNA of re-grown transconjugants was extracted for 16S rRNA sequencing. In total, transconjugants from the first time point (MS1) either re-grown in 10% soil extract (2 out of 4 replicates) or in 10% TSB after 10% soil extract (2 out of 4 replicates) were sequenced.

### DNA Extraction

All DNA extractions were performed with the NucleoSpin Soil kit (Macherey-Nagel; Germany), following the manufacturer’s instructions. Total DNA extracts were obtained from 250 mg of manured soil. In contrast, the DNA from the re-grown transconjugants was obtained after concentrating the cells by centrifugation (10,000*g*) and resuspension in 250 μL of sterile PBS. DNA quantification and a PCR reaction targeting 16S rRNA (466 bp amplicon size) were used to validate the DNA extractions and to confirm if there was significant growth of transconjugants. Only samples with a clear band at 466 bp compared to PCR negative controls (i.e., DNA extraction from the culture media and MiliQ water control in PCR) were further used for sequencing.

### 16S rRNA Sequencing

Amplicon sequencing libraries were prepared using a two-step PCR, targeting 16S rRNA gene V3-V4 regions. First PCR was performed for 30 cycles using the primers Uni341F (5′-CCTAYGGGRBGCASCAG-3′) and Uni806R (5′-GGACTACNNGGGTATCTAAT-3′) initially published by Yu et al.^35^ and modified as described by Sundberg et al.^36^ First PCR amplification products were purified using HighPrep PCR clean-up (MagBio Genomics) using a 0.65:1 (beads:PCR reaction) volumetric ratio. A second PCR reaction was performed to add Illumina sequencing adapters and sample-specific dual indexes (IDT Integrated DNA Technologies) using PCRBIO HiFi (PCR Biosystems Ltd., U.K.) for 15 cycles. The second PCR products were purified with HighPrep PCR Clean-Up System, as described for the first PCR. Sample concentrations were normalized using the SequalPrep Normalization Plate (96) Kit (Thermofisher), following the manufacturer’s instructions. The libraries were then pooled and up-concentrated using DNA Clean and Concentrator-5 Kit (Zymo Research). The library pool’s concentration was determined using the Quant-iT High-Sensitivity DNA Assay Kit (Life Technologies) and diluted to 4 nM. The library was denatured and sequenced following the manufacturer’s instructions on an Illumina MiSeq platform at the Section of Microbiology–University of Copenhagen, using Reagent Kit v3 [2 × 300 cycles] (Illumina).

Cutadapt v.2.3.^37^ was used to remove primer sequences used in the first PCR, both on the 5′ and the reverse complement on 3′ ends, also discarding read pairs for which any of the two primers could not be detected. Reads were further processed for error-correction, merging and amplicon sequence variants (ASVs) generation using DADA2 version 1.10.0^38^ plugin for QIIME2^39^ with the following parameters: truncL = 280, truncR = 240; trimL = 8, trimR = 8, and otherwise defaults parameters. Each ASV sequence was taxonomically annotated using *q2-feature-classifier classify-sklearn* module trained with SILVA SSU database version 132,^40^ trimmed for the V3-V4 region only.

Data analysis was performed using *phyloseq* version 1.22.3^41^ in R statistical software version 3.6.3^42^ and RStudio (Version 1.2.5033; https://www.rstudio.com/). Two datasets were created based on the sample’s origin. One consisted of the ASVs present in the manured soil microcosms (incl. original soil and manure), and the other of ASVs from the presumable transconjugants (sorted re-grown cells). In the microcosm and transconjugant datasets, ASVs were removed that were not assigned to Bacteria (*n* = 96 and *n* = 2, respectively), and assigned to chloroplasts (*n* = 8378 and *n* = 3, respectively) or mitochondria (*n* = 2851 and *n* = 5, respectively). Furthermore, using the *decontam* package,^43^ 14 predicted contaminant ASVs which were linked to blank controls (culture media extraction control, first and second PCR negative controls) were removed from the microcosm dataset, retaining a total of 50,244 ASVs across all samples (2,887,440 reads in total; 72,600 ± 21,575 reads per sample on average). The transconjugant dataset (re-grown sorted cells) was not subjected to analysis with the *decontam* package and contained 25 ASVs across all samples. The occurrence of ASVs in the controls was manually checked (Supporting Figure 4). Rarefaction curves and library sizes can be found in Supporting Figure 5. Phylogenetic trees were constructed using *phyloseq*. The raw reads can be accessed under the NCBI Bioproject number PRJNA718741.

### Alpha and Beta Diversities

Samples with less than 20,000 reads were excluded (one soil sample removed). For the overall microcosm bacterial community, alpha diversity indexes (Chao1 richness, Shannon, and Pielou’s evenness) were estimated after rarefaction (*n* = 32,254). The microcosms dataset consisted of 40,959 ASVs, distributed in 30 samples consisting of manure (2939 ASVs, three samples) and soil samples (38,952 ASVs). Rarefying at 32,254 reads resulted in 9285 ASVs removed from the dataset. Rarefaction was only performed to estimate the α diversity indexes of the microcosm dataset. No rarefaction nor diversity index calculations were performed for the transconjugant dataset.

Beta diversity analysis, using Bray–Curtis dissimilarities, was calculated using the R package *vegan.*(44) The effects of incubation time on microcosms were determined using permutational multivariate analysis of variance (PERMANOVA) and depicted in a nonmetric multidimensional scaling (NMDS) ordination plot based on Bray–Curtis distances with 999 permutations. The homogeneity of group dispersion was confirmed by testing for multivariate homogeneity of group dispersions (PERMDISP2).

### Statistical Analysis

One-way analysis of variance (ANOVA) was conducted to detect differences in bacterial diversity indexes and in cell abundances between temperatures and time points. The ANOVA tests were followed by TukeyHSD post hoc analysis, and homogeneity of variance was confirmed with Levene’s test. Data normality was verified with Shapiro–Wilk’s method, and when normality was not achieved, group comparison was performed using the equivalent nonparametric test (Kruskal–Wallis). A significance score of *p* < 0.05 was considered statistically relevant. These analyses were performed with R version 3.6.3^42^ and RStudio (Version 1.2.5033; https://www.rstudio.com/). Used software packages consisted of *reshape* (Wickham, 2007) and *tidyverse* (Wickham et al., 2019); a set of packages designed for data cleaning, trimming, and visualization; *PMCMRplus* (Thorsten, 2020), and *car* (Fox and Weisberg, 2019) for ANOVA and Levene’s test.

## Results

### Representativity of Manure Application on Overall Microcosm Soil Diversity

The diversity indexes of the soil microcosms were calculated to confirm that the changes provoked by manure application were similar to the ones observed in other studies. Ultimately, these results showed that what was expected to happen after manure application, indeed, happened, thus assuring the microcosms representativity. In turn, this validated the conditions in which the quantification of the conjugation events occurred.

**Figure 1:**
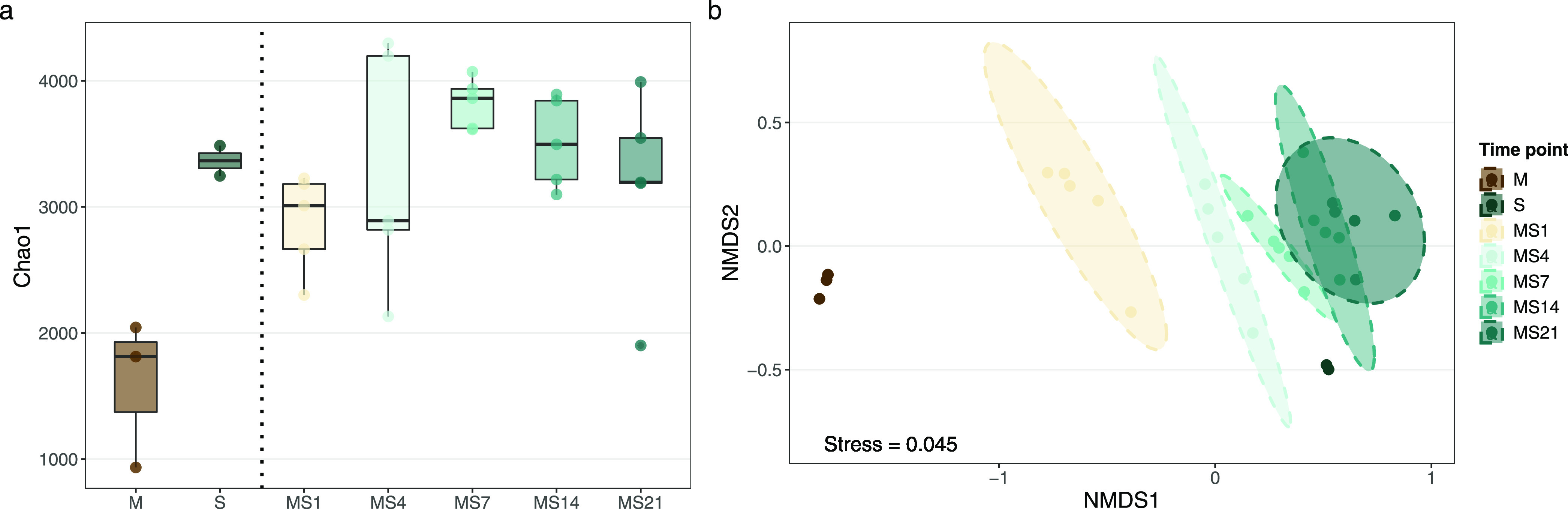
Manure application changed the bacterial community structure. Manure samples (M) had lower bacterial diversity than soil samples (S) and manured soils after days 1, 4, 7, 14, and 21 (MS1 to MS21, respectively) (a). Nonmetric multidimensional scaling (NMDS) plots illustrating Bray–Curtis dissimilarity matrices show clustering of the soil bacterial community samples by time after manuring (b), with the strongest shift seen right after manure amendment. The dataset presented in this figure was rarefied, as mentioned earlier. Other α diversity indexes can be found in Supporting Table 3.

Overall, Chao1 patterns showed that soil samples had a greater richness of bacterial ASVs than manure (Figure 1; *p* < 0.01, ANOVA), and application of manure at the relatively small proportion used (40 mg/g) did not increase the estimated total number of ASVs found in manured soils (Figure 1; *p* = 0.24, ANOVA) compared to the soil before manure application, respectively. Similar results have been reported in field studies elsewhere,^45−48^ and they corroborate that this treatment reflected field-level applications.

The NMDS ordination based on Bray–Curtis dissimilarity revealed clustering of samples according to time after manure application (Figure 1; PERMANOVA, *p* < 0.01). The effect of the time points explained 56% of the variation in the microcosm samples.

Before manure application, *Proteobacteria* (28.60 ± 0.01%), *Actinobacteria* (19.99 ± 1.32%), and *Acidobacteria* (14.15 ± 0.37%) were the most abundant *phyla* in soils. However, after manuring, *Proteobacteria* (45.60 ± 9.25%), *Bacteroidetes* (16.73 ± 4.64%), and *Firmicutes* (13.00 ± 2.58%) became dominant, and these were also highly abundant in manure (Supporting Figure 2).

### Quantification of Conjugation Events in Manured Soil Microcosms

**Figure 2:**
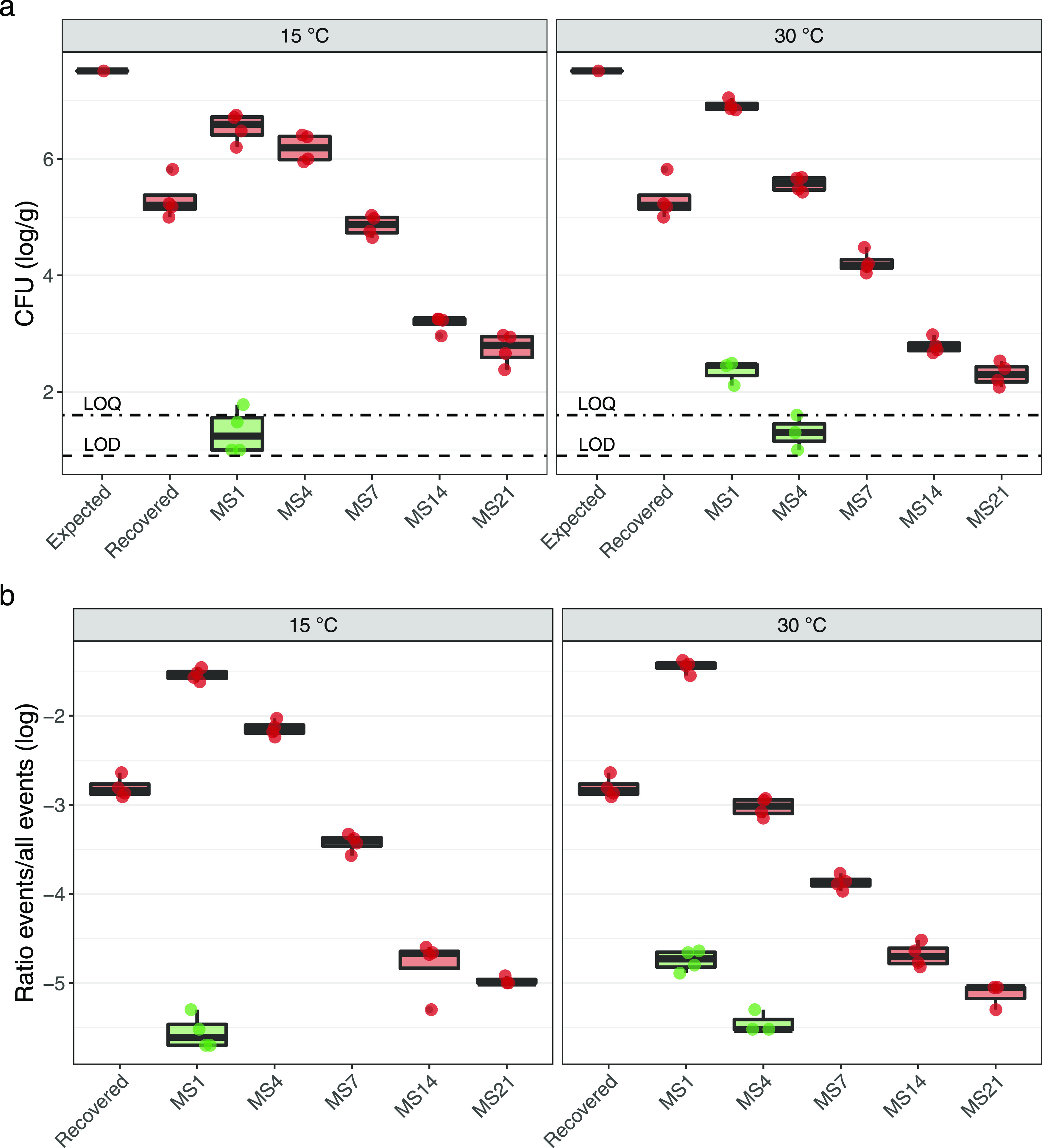
Transconjugants are detected shortly after manure application. Boxplots show the abundance of the donor (red) and transconjugants (green) in manured soil microcosms determined by plating (a) and flow cytometry (b). Colony-forming units (CFU) of donor and transconjugants were enumerated immediately after manure application (Recovered) and measured after incubation for 1, 4, 7, 14, and 21 days (MS1–MS21, respectively). Based on initial donor concentrations, 7.51 log CFU/g were spiked (Expected). Flow cytometry donor and transconjugant counts were normalized by events, and 1 × 10^6^ events were quantified per measure. The respective limit of quantification (LOQ, 1.6 log CFU/g) and limit of detection (LOD, 0.9 CFU/g) are also depicted.

Enumeration of donor and transconjugant by plating showed that transconjugants were only observed within the first four days of incubation (Figure 2), and at low abundance. On day 1 (MS1), transconjugants were observed in both temperatures (i.e., 15 and 30 °C), at 1.00–2.49 log CFU/g manured soil (equivalent roughly to 10^–5^–10^–4^ T/D; Supporting Table 1). On day 4 (MS4), transconjugants were only found at 30 °C and at lower abundances than in MS1 (*p* < 0.05, ANOVA; 1.00–1.60 log CFU/g manured soil, equivalent to 10^–5^–10^–4^ T/D; Supporting Table 1). No transconjugants were detected at MS4 in the 15 °C series.

The detection of transconjugants coincided with the peak of donor cells, whose abundances initially increased in both 15 and 30 °C series (*p* < 0.05), reaching 6.20–7.05 log CFU/g manured soil (MS1) and decreased since then (*p* < 0.05) to 2.08–2.97 log CFU/g manured soil (MS21) (Figure 2). The donor recovery, considered as the difference between observed (recovered) and predicted (expected) abundance, was low (−2.20 ± 0.36 logs). Based on initial inoculum concentration (8.90 logs CFU/mL), it was predicted to detect 7.51 log CFU/g after consideration of all dilution and Nycodenz extraction steps, but only 5.31 ± 0.36 log CFU/g were found (Supporting Table 1).

Remarkably, the results of flow cytometry resembled the results obtained with plating, with average transconjugant-to-donor ratios (T/D) reaching −5.22 ± 0.23 logs (plating) and −4.01 ± 0.16 logs (flow cytometry), at MS1 (Supporting Table 1 and 2). Although the majority of bacteria are known to be nonculturable,^49^ the plating was included to provide absolute concentrations of transconjugants per g soil.

### Identification of the New Plasmid Hosts (i.e., Transconjugants)

The criterion for naming “transconjugant” was generally based on the combination of growth in the selective media with green fluorescence, as this was indicative that the tagged plasmid was acquired. However, for the cell sorting, only the fluorescence was considered, like in established procedures.^25,28,29^ The ASV relative abundances were calculated by dividing the number of reads corresponding to that ASVs by the sum of the reads in the sample (Supporting Table 4).

**Figure 3:**
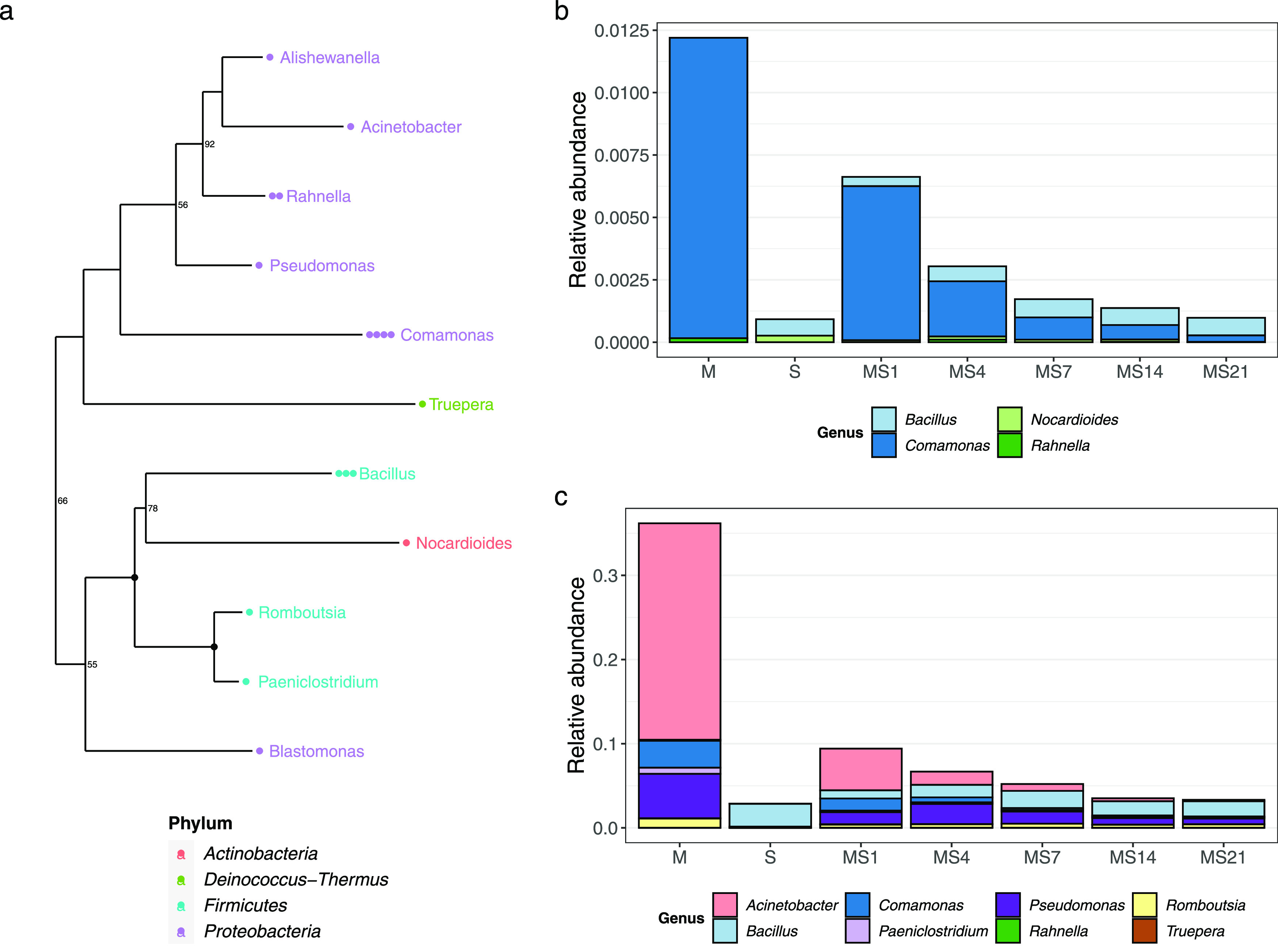
Overview of transconjugant bacterial genera and corresponding relative abundance in the microcosms. The phylogenetic tree shows the transconjugant genera found (a). Bar charts show the replicate-averaged relative abundance of (b) the ASVs of the transconjugants that were also detected in the microcosms, original soil, manure, and relative abundance of (c) the genera of the ASVs identified in the transconjugant pool. The average relative abundance of each genus is depicted in manure samples (M), soils (S), and manured soils on days 1, 4, 7, 14, and 21 (MS1 to MS21, respectively).

In the transconjugant pool, 19 ASVs were identified after excluding the ASVs co-detected in control samples (e.g., blank extractions; Supporting Figure 4), and these represented four major bacteria phyla in a total of 11 families and 11 genera (Figure 3a). An overview of the bacterial genera identified in the controls of the transconjugant pool can be found in Supporting Figure 6, and the relative abundance of the ASVs found in the controls in microcosms can be found in Supporting Figure 7.

Both Gram-positive (*Bacillus*) and Gram-negative (*Acinetobacter* and *Comamonas*) were among the most frequently detected genera among transconjugants. From the 19 transconjugant ASVs, only four were directly detected in the total manure/soil community. These are referred to *Bacillus* and *Nocardioides*, and *Comamonas* and *Rahnella* (Figure 3b). The ASVs from *Comamonas* were detected in manure and soils after, but not before manure application. Once introduced to soils, their relative abundance decreased over time (*p* < 0.05; Figure 3b and Supporting Table 4). In contrast, the other 15 ASVs were not detected at any time point in the total manure/soil community.

While part of the ASVs identified in the transconjugant pools was not found in manure or soils (15 out of 19 ASVs), the genera to which these ASVs corresponded were searched in the total manure/soil community (i.e., microcosm dataset) to determine their probable source. These genera were mainly found in manure and included *Acinetobacter*, *Pseudomonas*, and *Romboutsia* (Figure 3c).

## Discussion

In this study, we hypothesized that quantifiable conjugation events with a fecal commensal bacterium (*E. coli*) as donor would occur in manured soil microcosms and that indigenous soil bacteria would be identified among the taxa carrying the transferred plasmid. The results confirmed that not only did plasmids in manure bacteria conjugate in a manured soil context, but that native soil bacteria were able to acquire the plasmid.

### Manure Bacteria Conjugate in Manured Soils

The maximum number of transconjugants in this study corresponded roughly to a transconjugant-to-donor ratio (T/D) of 10^–4^ (transconjugant abundance of 10^2^ and 10^3^ CFU/g soil), which is similar to ratios found in soils in the literature. In early sterile soil studies using *E. coli* as donor, the T/D ratio varied between 10^–2^ and 10^–4^ (transconjugant abundance of 10^2^ and 10^3^ transconjugant CFU/g soil, respectively),^15,16^ and Top et al. reported conjugation ratios of 10^–4^ T/D with an IncP1 plasmid (corresponding to 10^2^ transconjugant CFU/g soil) in nonsterile soil, but only when nutrients were added. On the other hand, disparate transconjugant abundances have also been reported. The diversity of experimental setups can partly explain the high variability of observed transfer frequencies among studies. Besides the individual donor, recipient, and vector characteristics, most studies were performed under scenarios that do not adequately simulate the complexity found in the environment (e.g., sterile soils, filter mating, nutrient-rich media). Several factors may affect the plasmid transfer frequency in soils, and caution is advised when comparing values between studies. However, despite the variability in observed transconjugant abundance, the findings of the present study, conducted under more complex conditions, are consistent with the findings of the published literature.

The maximum number of transconjugants was obtained shortly after manure application (within the first four days). Similar findings have been reported by,^14^ where transconjugants were mainly found shortly after introducing *E. coli* donor strains in soil microcosms. However, depending on the soil type, these results were mainly achieved after the introduction of nutrients. Elsewhere, manure application to soils was responsible for a 10-fold increment of transconjugants.^50^ Conjugation is known to require energy and cell resources,^51−53^ and it has been shown that conjugation rates depend on nutrient availability,^54^ and nutrient availability is a known factor influencing bacterial survival in soils.^18^ Additionally, this study also shows that the days immediately after manure application are likely to be critical to the plasmid transfer of manure-associated donors, and more research should be conducted to address the variations in soil conjugation rates shortly after manure has been applied.

In this study, conjugation occurred under more realistic environmental conditions but at similar moderate rates to previously reported experiments. However, donors were spiked in larger concentrations than typically present in manure. This was done due to methodological implications, but we suspect it would still happen at lower concentrations, although below our detection limits. Overall, in the Netherlands alone, over 76 million tons of animal manure are produced every year, most of which is applied untreated on farmlands.^55^ Manure typically contains 10^5^ CFU/g of *E. coli,*(56) which results in the application of roughly 10^15^*E. coli* CFU to the roughly 1.1 million hectares of grassland (CBS StatLine; https://opendata.cbs.nl/statline/#/CBS/en/). Despite conjugation occurring at low frequencies, the scale of manure application is sufficiently frequent to enable a large number of potential transfer events. However, while IncP1 plasmids are abundant in the environment,^57^ they are not so common among *Enterobacteriaceae*, and consequently, are not representative of plasmid families known for their AMR carriages such as IncF, IncI, IncA/C, or IncH.^58^ As shown here, environmental conditions and farming practices may promote conditions for conjugation. In the Netherlands, manure may only be applied between February and August/mid-September when the topsoil temperature is higher.^59^ As seen in this study, higher temperatures may result in more transconjugants, and more transconjugants may imply longer plasmid persistence in the bacterial community. Additionally, farmers typically apply manure to soils multiple times per season. While the present study only simulated one manure application, it is possible that transconjugants accumulate if multiple manure events occur within a short time frame. For example, it has been shown that manure provokes an intense short-term increase of ARGs (after four days) in manured soils, which would generally decrease after a couple of weeks.^45,60^ On the other hand, it has been reported that the abundance of selected ARGs at the beginning of a new manure application round was higher than at the beginning of the first manuring round, roughly 40 days after the first application.^61^ Ultimately, as manure contains many features favorable to HGT,^17^ multiple manure applications in a short time frame can lead to the accumulation of ARGs and plasmid-carrying bacteria, either by direct input, or because it may provide the nutrients needed to compensate for eventual plasmid fitness or acquisition costs in the transconjugants. Therefore, this study suggests that several requirements and conditions that might facilitate plasmid acquisition in the soil bacterial community may already be fulfilled.

### Manure Application Led to Plasmid Uptake from Fecal Donors by Native Soil Bacteria

Overall, manure application to soils resulted in the detection of transconjugants, from which several genera were also identified in soil or manure. Among the transconjugants, ASVs from *Bacillus* and *Nocardioides* were traced back to soils. These genera are ubiquitous and thus commonly found in soils. The presence of members of the order *Bacillales* and the genus *Nocardioides* among transconjugants has been reported in some soil community permissiveness studies,^25,28^ but not in all.^26,27^ Recently, both *Bacillus* and *Nocardioides* have been found in the transconjugant pool of soil microcosms after 5 and 75 days of incubation,^31^ suggesting that maintenance of the acquired plasmid is possible. However, that was not observed in the present study. Although the relative abundance of these two genera remained relatively constant throughout the experimental time frame, no transconjugants were detected after four days of incubation. Depending on the context, the acquisition of a new plasmid may promote bacterial survival but also reduce the fitness of the plasmid-carrying host due to an increased metabolic burden.^62,63^ Nevertheless, it is relevant that native soil bacteria can acquire ARG-carrying plasmids from a manure-specific donor, as demonstrated in the current study.

This study used a conjugative IncP1 plasmid, which is considered to be mostly environmental. IncP1 plasmids have been found in high abundances in manure^64^ and soils,^57^ and they were reported to carry genes conferring resistance to multiple antibiotics (e.g., β-lactams, sulfonamides, aminoglycosides, and tetracyclines).^58^ As the name implies, broad-host-range plasmids can be transferred between distinct phylogenetic groups of bacteria, explaining the diversity of bacterial phyla observed among transconjugants. *Acinetobacter* and *Pseudomonas* are common environmental bacteria and have been consistently found in the recovered soil transconjugant pool.^4,25,26,28,31^ However, in this study, most of the ASVs corresponding to these genera were not further detected in the manured soils. This may be due to abundances below the detection limit. Because the overall number of sorted cells was low and required a re-growth step before sequencing, it is possible that these ASVs were too rare to be detected in the more diverse bacterial community of manure and soil. By tracing the genera in the microcosms, instead of the specific ASVs, it was shown that most of these genera were more abundant in manure than in soil, which was possibly their source.

### Limitations of the Experimental Setup

To some extent, all methodologies imply a certain level of bias, including methodologies applied in this study which set out to quantify HGT at levels around the methodological detection limit. Such bias could lead to an underestimation of conjugation events or of the bacterial taxa receiving the plasmid. For example, performing Nycodenz extraction has been shown to result in underrepresentation of *Firmicutes* and *Actinobacteria.*(65) However, genera belonging to these *phyla* were found among the transconjugant pool (i.e., *Bacillus* and *Nocardioides*). Previous studies using a similar approach were able to demonstrate that transconjugants include a wide range of species soil bacterial communities.^25,27^ While it is possible that other relevant taxa were not detected because of the chosen approach, we demonstrated that manure-introduced plasmids were acquired by native soil bacteria when manure was applied.

The amount of *E. coli* added (10^7^ CFUs/g soil) was larger than in realistic field-scale manure amendments, based on *E. coli* soil concentrations resulting from manure amendment in field situations equaling 10^5^ CFUs/g soil.^56^ However, compared to the amount of total bacteria typically added with manure in field situations (resulting soil concentrations of 10^7^–10^8^ 16S copies/g soil^61^), an addition of 10^7^ CFUs/g soil is comparable.

The potential influence of natural transformation and transduction was not considered in this study, and, consequently, cannot be excluded as a possible cause. Bacteriophages are significant ARG reservoirs^66,67^ and can also be abundant in cattle manure^68^ and in soils.^69^ However, their numeric contribution to HGT is not clear. Furthermore, ARGs in the bacteriophage fractions are found at concentrations roughly 10-fold lower or less than in the corresponding bacterial fraction.^70,71^ The uptake of extracellular DNA (exDNA) by natural transformation is another one of several ways bacteria can acquire new genetic information given sufficient size, concentration, and integrity of the DNA.^72^ Natural transformation is known to lead to the acquisition of ARGs^73^ and mobile genetic elements,^74,75^ but soil matrices may have inhibitory effects on transformation and exDNA availability,^76^ and the stability of the exDNA in soil microcosms may vary from hours to days.^72^ While ARG transfer may also occur through transformation or transduction, in this study, we focused on conjugation because it is often considered the most likely responsible mechanism for ARG transfer.^67^

Regarding the plate counts, plating was initially seen as a fallback option to directly enumerate and identify transconjugants. Because the majority of bacteria are known to be nonculturable,^49^ the results obtained with flow cytometry were expected to be more representative of the conjugation events occurring in the microcosms than the plate counts. However, the results were remarkably similar, hinting that the majority of the transconjugants observed in flow cytometry could be cultured if needed.

Additionally, due to low transconjugant numbers observed with flow cytometry, it was not possible to sequence the transconjugant pool community immediately. To overcome this challenge, another bias was introduced by regrowing the sorted transconjugants in diluted culture media. First, soil extract was used as, presumably, it would maintain nutritive conditions similar to the ones the soil bacteria would be adapted to. After failing to promote visible growth, diluted TSB broth was used to provide sufficient but not excessive nutrients. Nevertheless, to accommodate for this, the manure-soil microcosms were also sequenced directly. The sequencing of the transconjugant pool was used only to identify the bacterial groups, and the relative abundances shown in Figure 3(b,c) were obtained by combining the bacterial group identities (i.e., ASVs) with the overall microcosms’ community. Therefore, the impact of regrowing the transconjugants is expected to be low.

In this microcosm study, *E. coli* representing fecal bacteria successfully transferred a broad host range plasmid to soil and manure bacteria via conjugation. Despite occurring at low frequencies, HGT was observed until the first 4 days after manure application. Among the new plasmid hosts (transconjugants), *Bacillus* and *Nocardioides* were linked to soils and *Comamonas* and *Rahnella* were linked to manure. *Acinetobacter* and *Pseudomonas* were identified in the transconjugant pool, but their abundance was probably below the detection limit, as it was not possible to track their specific ASVs in the microcosms.

This study shows that despite constraints posed by environmental conditions such as nutrient and temperature, manure amendment might result in conditions enabling ARG-carrying plasmid transfer from manure to the soil bacterial community. However, transconjugants did not thrive after 4 days of the experiment, indicating that other factors not evaluated here may play a role in hampering the colonization of the plasmids in the new hosts. Further understanding of those factors and how they affect the fate of ARG vectors is needed, but the current study already provides important information to determine the risks of AMR spread via manure application on land.

## Supporting Information Available

Abundances of donor and transconjugants obtained by plating (Table 1); abundances of donor and transconjugants obtained by flow cytometry (Table 2); sample diversity indexes (Table 3); ASV relative abundance of *Bacillus*, *Comamonas*, *Nocardioides*, and *Rahnella* in samples (Table 4); genetic map of donor (*E. coli* MG1655) and plasmid (pKJK5) (Figure 1); relative abundance of the main bacteria *phyla* found (Figure 2); sample processing workflow (Figure 3); Venn diagram of the number of ASVs found (Figure 4); rarefaction curves obtained from sequencing (Figure 5); dendrogram showing the genera found in the control samples (Figure 6); and relative abundance of control ASVs tracked in the microcosms (Figure 7) (PDF)
Details of samples (XLSX)
