# Supplementary material for: Horizontal Gene Transfer of an IncP1 Plasmid to Soil Bacterial Community Introduced by Escherichia coli through Manure Amendment in Soil Microcosms
Source: Environ Sci Technol. 2022 Jul 27;56(16):11398–408. doi: 10.1021/acs.est.2c02686 (PMC9387108; doi:10.1021/acs.est.2c02686)
Supplement: Supplementary file 1 — es2c02686_si_001.pdf [file es2c02686_si_001.pdf]

Supporting information

Horizontal gene transfer of an IncP1 plasmid to soil bacterial community introduced by *Escherichia coli* through manure amendment in soil microcosms

Gonçalo Macedo[a,b]*, Asmus K. Olesen[c], Lorrie Maccario[c], Lucia Hernandez Leal[b], Peter v. d. Maas[d], Dick Heederik[e], Dik Mevius[a,f], Søren J. Sørensen[c], Heike Schmitt[b, e, g]

Affiliations:

[a] Department of Infectious Diseases and Immunology, Faculty of Veterinary Medicine, Utrecht University, Yalelaan 1, 3584 CL Utrecht, The Netherlands

[b] Wetsus, European Centre of Excellence for Sustainable Water Technology, Oostergoweg 9, 8911 MA Leeuwarden, The Netherlands

[c] Department of Biology, University of Copenhagen, Copenhagen, 2100, Denmark

[d] Van Hall Larenstein, University of Applied Sciences, Agora 1, 8901 BV Leeuwarden, The Netherlands

[e] Institute for Risk Assessment Sciences, Utrecht University, Yalelaan 2, 3584 CM Utrecht, The Netherlands

[f] Department of Bacteriology and Epidemiology, Wageningen Bioveterinary Research, Houtribweg 39, 8221 RA Lelystad, The Netherlands

[g] Centre for Infectious Disease Control, National Institute for Public Health and the Environment (RIVM), Antonie van Leeuwenhoeklaan 9, 3721 MA Bilthoven, The Netherlands

* Corresponding author: g.n.barrocademacedo@uu.nl

Pages: 12

Tables: 4

Figures: 7

Supplementary Table 1. Donor and transconjugant abundance in soil microcosms, obtained by plating.

Temperature	Time point	Donor		TC		T/D	
		Mean	SD	Mean	SD	Mean	SD
15 °C	Recovered	5.31	0.356				
	MS1	6.54	0.253	1.32	0.384	-5.22	0.227
	MS4	6.18	0.244				
	MS7	4.86	0.180				
	MS14	3.17	0.142				
	MS21	2.74	0.276				
30 °C	Recovered	5.31	0.356				
	MS1	6.92	0.095	2.35	0.209	-4.52	0.248
	MS4	5.56	0.129	1.30	0.300	-4.29	0.427
	MS7	4.22	0.187				
	MS14	2.79	0.136				
	MS21	2.30	0.201				

Notes: colony forming units (log CFUs/g soil) were enumerated for donors and transconjugants (TC) at multiple time points: immediately after manure application (Recovered), and after incubation at day 1, 4, 7, 14, and 21 (MS1 – MS21, respectively). For the donors and TC, the values in the table were obtained by calculating the mean and standard deviation (SD) of the four replicates of each time point. The transconjugant-to-donor ratios (T/D) were calculated by dividing the TC mean value by the donor measured in that time point. All values are presented in log_{10}.

Supplementary Table 2. Donor and transconjugant abundance in soil microcosms, obtained by flow-cytometry.

Temperature	Time point	D/E		T/E		T/D	
		Mean	SD	Mean	SD	Mean	SD
15 °C	Recovered	-2.81	0.119				
	MS1	-1.54	0.069	-5.56	0.19	-4.01	0.158
	MS4	-2.14	0.089				
	MS7	-3.43	0.103				
	MS14	-4.81	0.328				
	MS21	-4.97	0.046				
30 °C	Recovered	-2.81	0.119				
	MS1	-1.45	0.073	-4.75	0.119	-3.30	0.147
	MS4	-3.03	0.105	-5.45	0.127	-2.39	0.181
	MS7	-3.87	0.083				
	MS14	-4.69	0.135				
	MS21	-5.13	0.144				

Notes: for each time point, red and green counts were measured by flow-cytometry. The donor-to-events (D/E) and transconjugant-to-events (T/E) were obtained by dividing the red (donors) or green (transconjugants) counts by the total number of events recorded (1×10^6 events). The values in the table represent the log mean and standard deviation (SD) of the four replicates of the time points. The transconjugant-to-donor (T/D) ratios were calculated by dividing the green (transconjugants) with the red (donors) counts. All values are logarithmic with base 10 (log_{10}).

Supplementary Table 3. Averaged alpha diversity indexes, and corresponding standard deviations, of manure (M), soil (S), and manured soils at days 1, 4, 7, 14, and 21 (MS1 to MS21, respectively), grouped by time points. This microcosm series was incubated at 30 °C.

Timepoint	Observed		Chao1		Shannon		Simpson		Evenness	
	Mean	SD	Mean	SD	Mean	SD	Mean	SD	Mean	SD
M	1420	432	1597	586	5.501	0.234	0.9779	0.0035	0.7618	0.0070
S	2975	54	3366	170	7.217	0.025	0.9986	0.0000	0.9023	0.0011
MS1	2544	229	2877	390	6.041	0.516	0.9498	0.0274	0.7711	0.0719
MS4	2890	611	3267	943	7.084	0.168	0.9980	0.0005	0.8912	0.0143
MS7	3259	126	3821	200	7.202	0.071	0.9981	0.0006	0.8904	0.0067
MS14	3021	236	3509	358	7.121	0.080	0.9983	0.0003	0.8890	0.0096
MS21	2750	506	3164	779	7.024	0.183	0.9980	0.0004	0.8888	0.0048

Supplementary Table 4. Relative abundance of the ASVs identified as *Bacillus*, *Comamonas*, *Nocardioides*, and *Rahnella* in manure (M), soil (S), and manured soils at days 1, 4, 7, 14, and 21 (MS1 to MS21, respectively).

Timepoint	Replicate	Bacillus	Comamonas	Nocardioides	Rahnella
M	A	0	0.007768297	0	0.000489342
M	B	0	0.019130325	0	0
M	C	0	0.009210292	0	0
S	A	0.000456858	0	0.000204799	0
S	B	0.00085093	0	0.000327281	0
MS1	A	0.000285232	0.004540891	0.000136911	0
MS1	B	0.000403086	0.00592119	0.00083397	0
MS1	C	0.000277204	0.006542017	0	0
MS1	D	0.000376514	0.005924564	0.000177183	0
MS4	A	0.000522687	0.002491103	0.000211299	0.00022242
MS4	B	0.000311515	0.002675365	0.000439786	0
MS4	C	0.000612908	0.001430118	0	0
MS4	D	0.000489441	0.002363643	0	0.000274565
MS7	A	0.000818531	0.000513109	0	0
MS7	B	0.000670098	0.00171545	0	0
MS7	C	0.000783387	0.000771335	0.000241042	0
MS7	D	0.000572233	0.001030019	0.000160225	0
MS14	A	0.000631525	0.001110262	0	0.000091673
MS14	B	0.000700744	0.000648837	0	0
MS14	C	0.000709595	0.000168951	0.000214005	0
MS14	D	0.000473833	0.000556753	0	0
MS21	A	0.000645578	0.000345845	0	0
MS21	B	0.000737657	0.00051636	0	0
MS21	C	0.000574143	0.000417558	0.0000835117	0
MS21	D	0.000682086	0	0	0

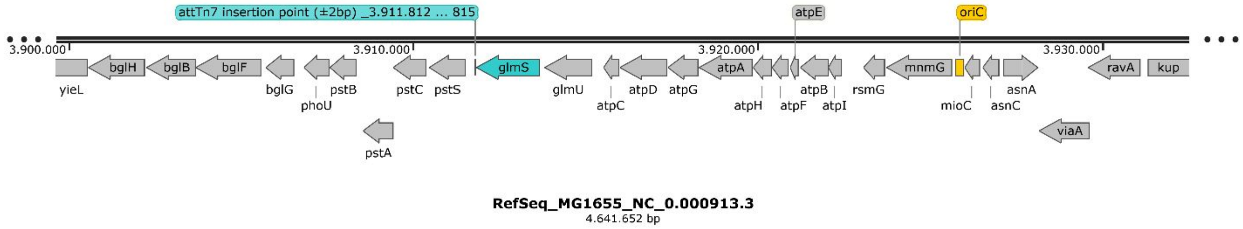

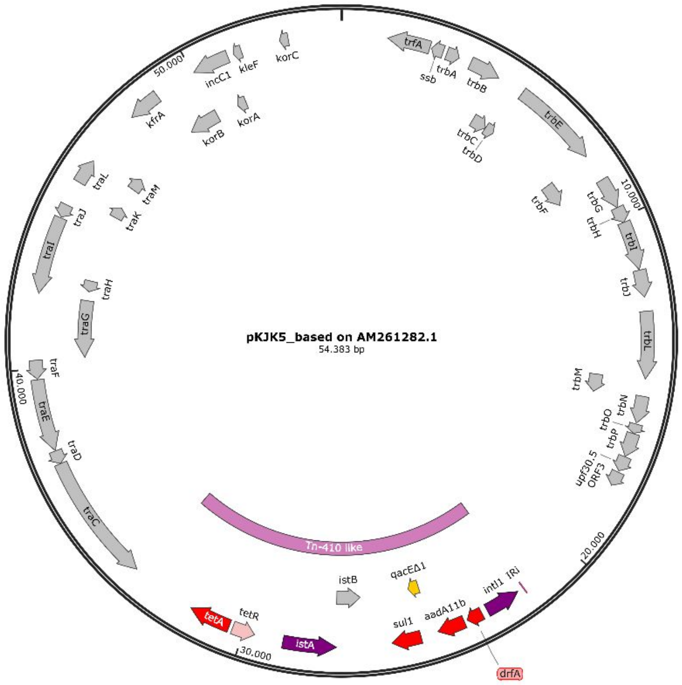

Supplementary Figure 1. Annotated genetic map of *E. coli* MG1655 (donor) and the conjugative pKJK5 plasmid. In the donor cell (Acc: NC_000913, top), the specific integration of the lacIq-pLpp-mCherry-Km^{R} gene cassette was performed in the chromosomal attTn7 site. In the pKJK5 plasmid (Acc: AM264282, bottom), the insertion of the Plac::gfpmut3b::Km^{R} was performed in the Tn-402 like element. The original pKJK5 plasmid is Tmp^{R} and Tet^{R}, but after the insertion of the gfpmut3b construct that is carried in a Km^{R} entranceposon, the resulting plasmid became Tmp^{R}, Tet^{S} and Km^{R}. These results were confirmed by the antibiotic susceptibility tests described in the main body of this manuscript. Illustration made by Rebeca Pallares-Vega using SnapGene software (from Insightful Science; available at snapgene.com). Details on the donor and plasmid tagging can be found in (Bahl et al., 2009; Klümper et al., 2015).

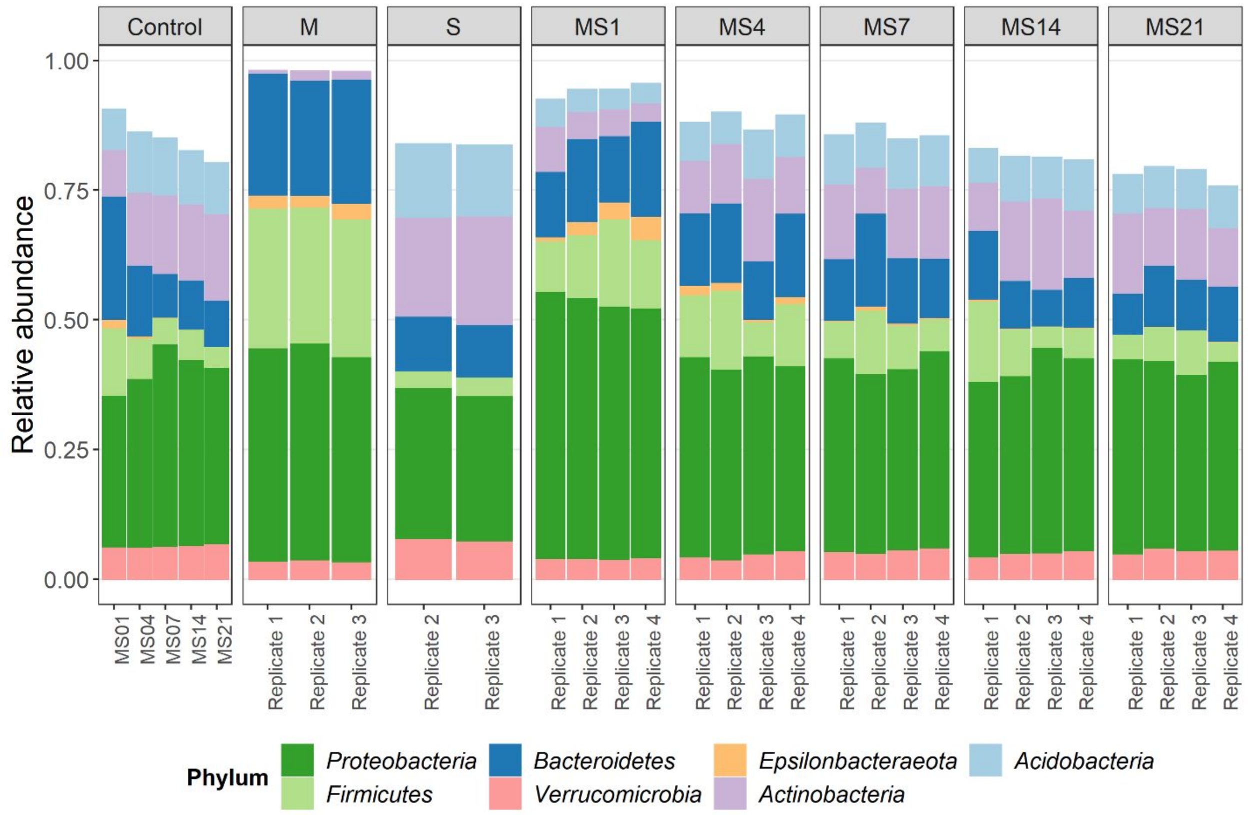

Supplementary Figure 2. The main *phyla* found in manure became the most abundant in soils after manure application. Bar charts showing the relative abundance in manure samples (M), in soils (S), and in manured soils at days 1, 4, 7, 14, and 21 (MS1 to MS21, respectively). The control series (i.e., manured soil without the donor spike) is also displayed. The control series (i.e., manured soil without the donor spike) is also displayed.

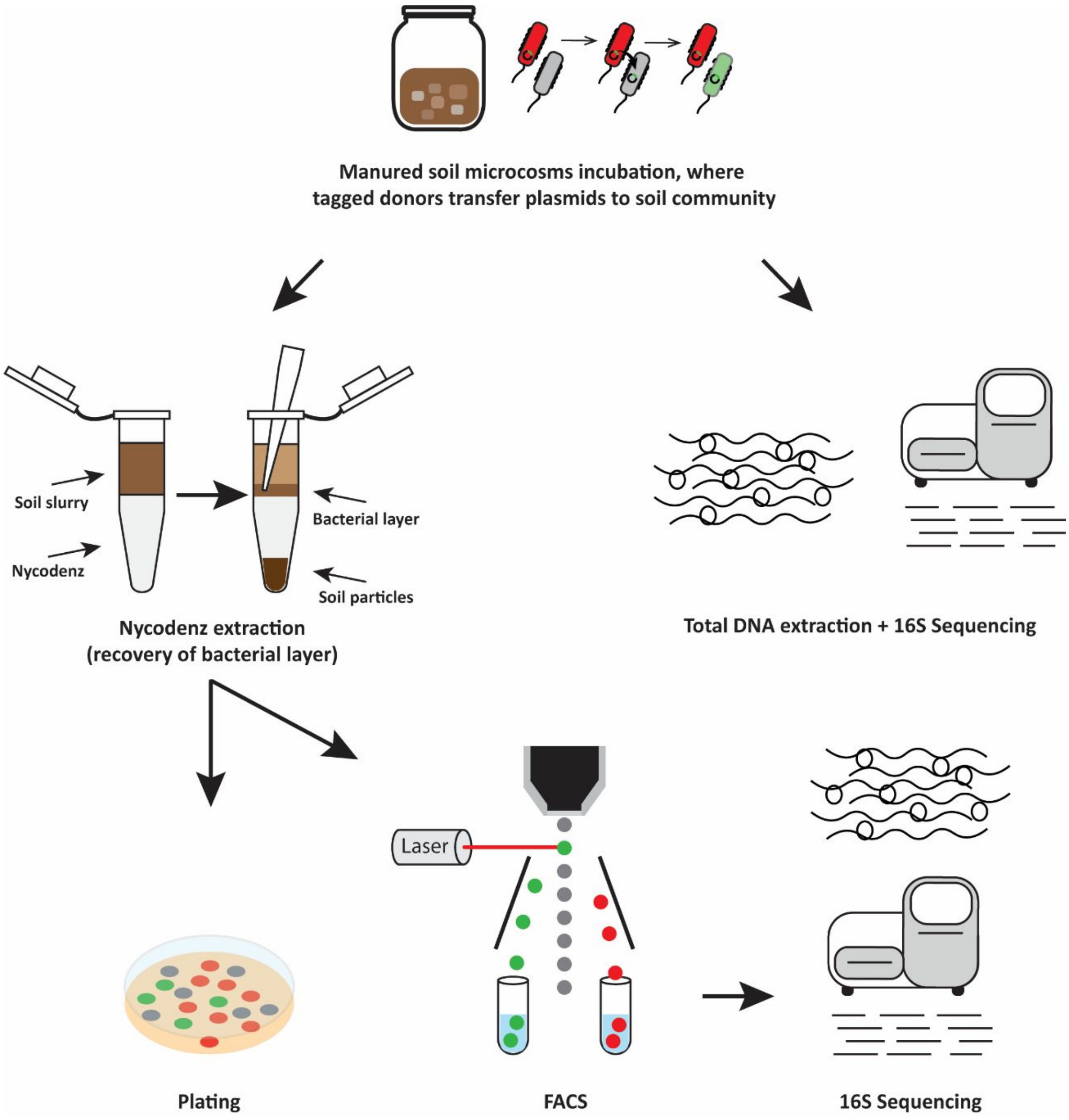

Supplementary Figure 3. Sample processing workflow. After incubation, the bacterial community from each microcosm was sequenced by 16S rRNA sequencing, and the soil slurry was also used for Nycodenz extraction. The bacterial layer of the Nycodenz extraction was then i) plated and ii) submitted to flow-cytometry and fluorescence-activated cell sorting (FACS). After FACS, the transconjugant pools were enriched and sequenced. For details on the mechanics involved in the transfer reporter-gene approach used to track conjugation events, the reader is directed to the publication by Sørensen et al. (2005).

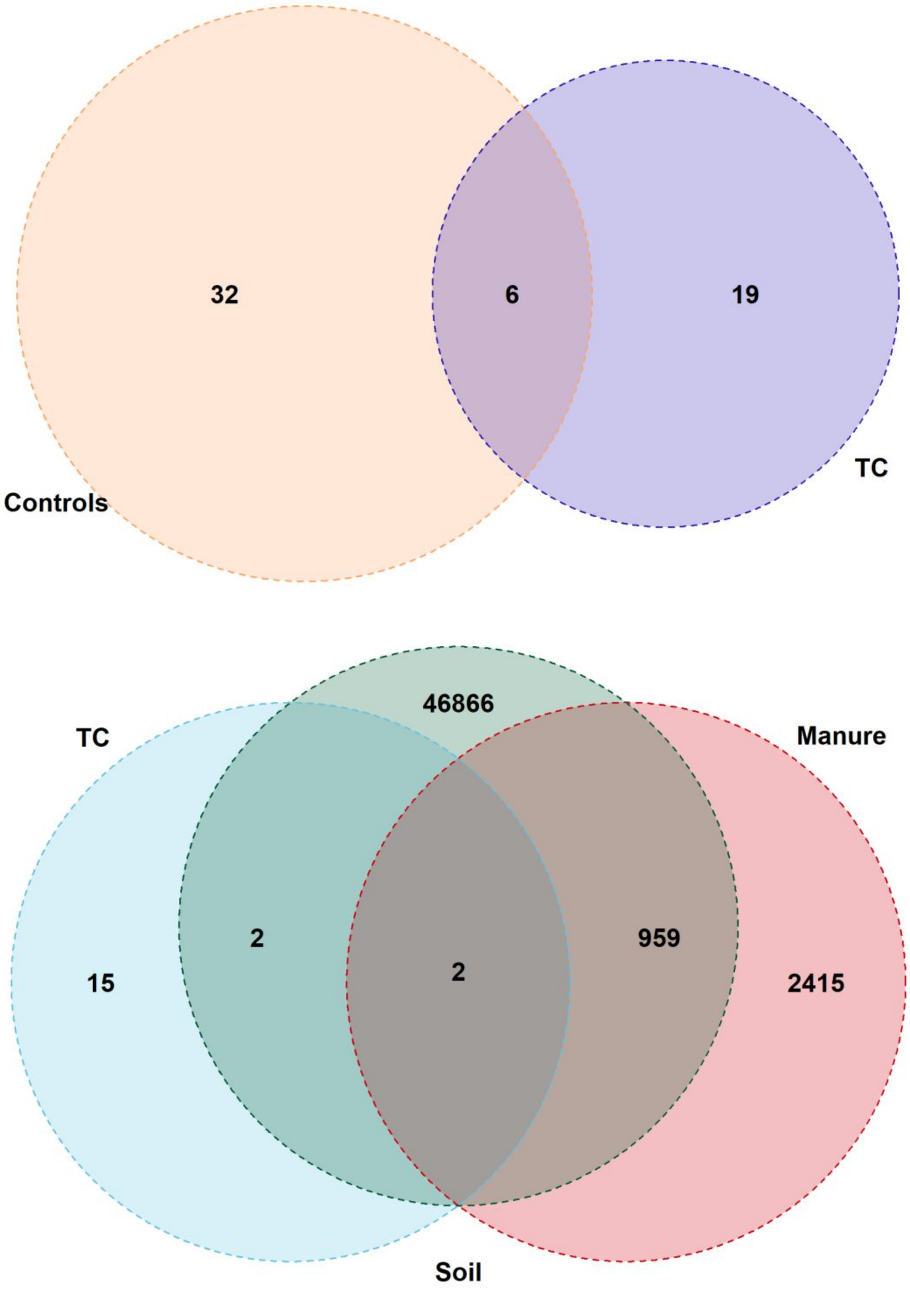

Supplementary Figure 4. Transconjugant ASVs found in controls were excluded from analysis. Venn diagram showing the number of ASVs found in the control samples and in the samples with transconjugants (TC). The group "controls" refers to a combination of ASVs in the media used for re-growth and the blank extractions.

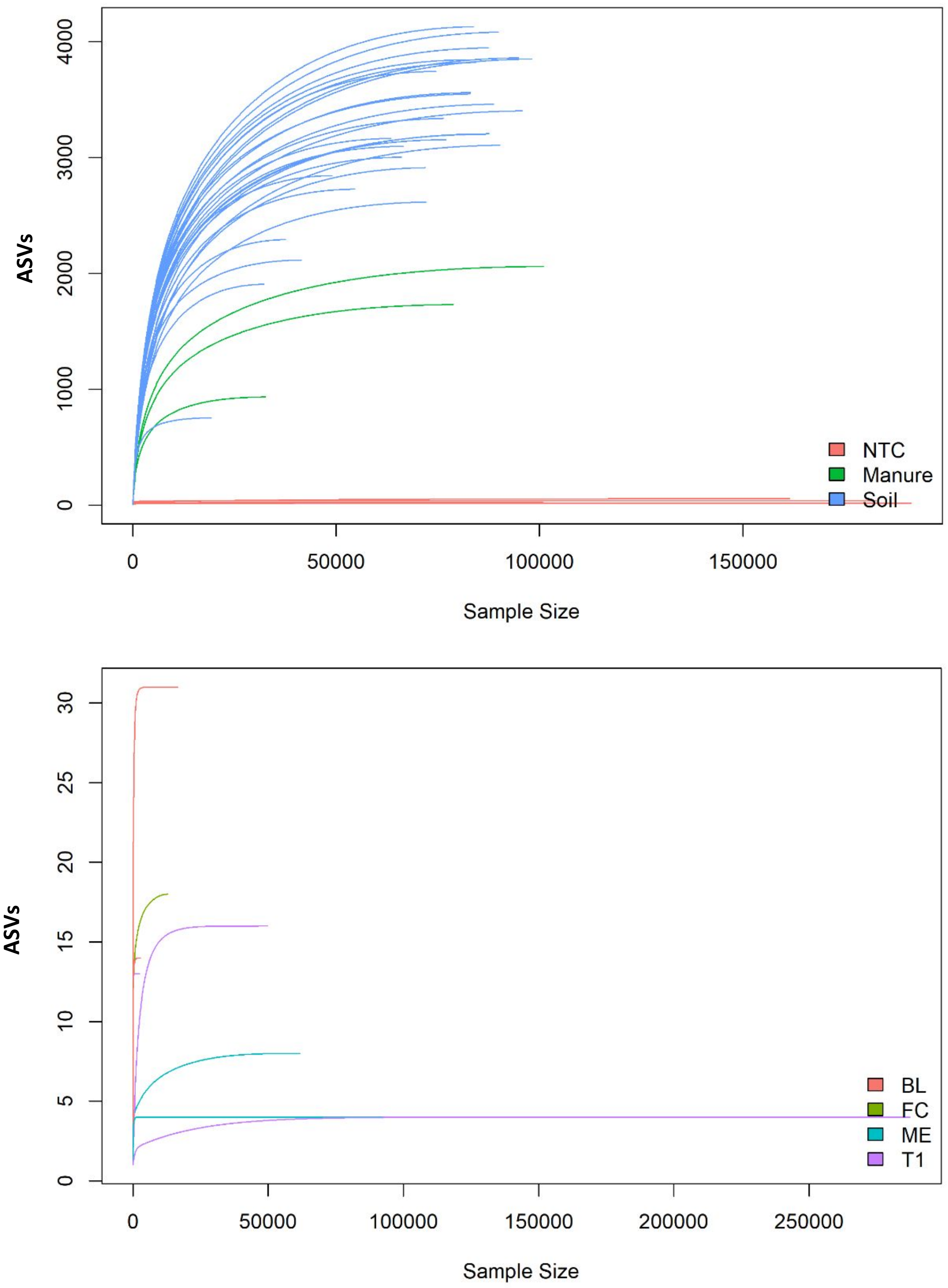

Supplementary Figure 5. Rarefaction curves show high ASV diversity in the microcosms (top), but not in the transconjugant dataset (bottom). In the latter, the ASVs found in samples obtained from re-grown sorted transconjugants (MS1) were compared with the in-line flow cytometry stream (FC) and with the media used for re-growth (ME). In both sequencing runs, blank extractions were included as additional controls (NTC and BL).

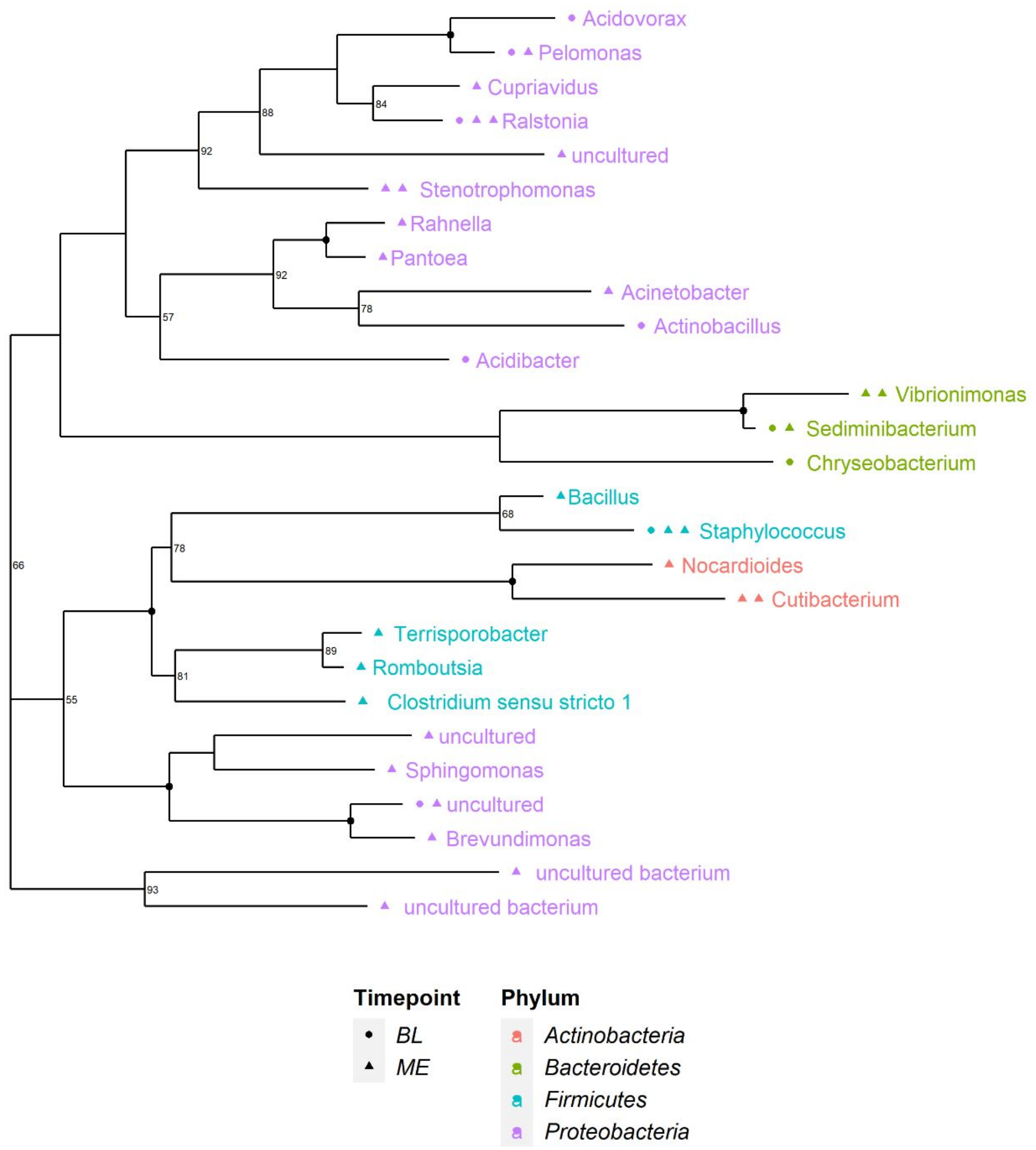

Supplementary Figure 6. Overview of the bacterial genera found in the controls of the transconjugant dataset. Dendrogram showing the genera found in the controls obtained from the in-line flow-cytometry stream (FC), the media used for re-growth (ME), and the blank extractions (BL). The dendrogram was created using the *phyloseq* package with bootstrap values displayed in the nodes.

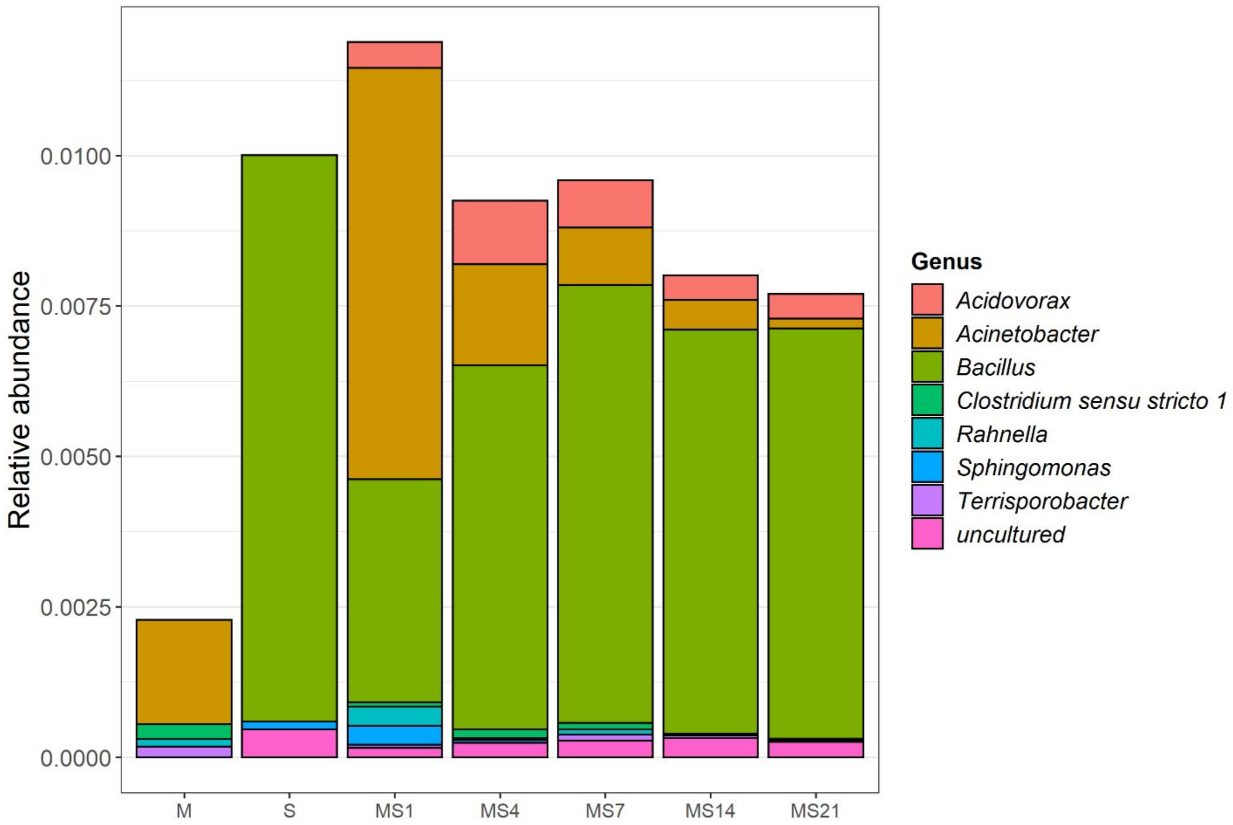

Supplementary Figure 7. Relative abundance of control ASVs tracked in the microcosms. Bar plot showing the relative abundance of the genera found in manure samples (M), in soils (S), and in manured soils at days 1, 4, 7, 14, and 21 (MS1 to MS21, respectively).
